# Challenges of Controlling Foot-and-Mouth Disease in Pastoral Settings in Africa

**DOI:** 10.1155/2024/2700985

**Published:** 2024-02-08

**Authors:** Mkama M. Mashinagu, Philemon N. Wambura, Donald P. King, David J. Paton, Francois Maree, Sharadhuli I. Kimera, Mark M. Rweyemamu, Christopher J. Kasanga

**Affiliations:** ^1^Department of Microbiology, Parasitology and Biotechnology, College of Veterinary Medicine and Biomedical Sciences, Sokoine University of Agriculture, P.O. Box 3019, Morogoro, Tanzania; ^2^SACIDS Foundation for One Health, Africa Centre of Excellence, P.O. Box 3297, Morogoro, Tanzania; ^3^Tanzania Veterinary Laboratory Agency, Centre for Infectious Diseases and Biotechnology, P.O. Box 9254, Dar es salaam, Tanzania; ^4^FAO World Reference Laboratory for FMD, The Pirbright Institute, Ash Road, Pirbright, Woking GU24 ONF, UK; ^5^Clinomics, Uitzich Road, Bainsvlei, Bloemfontein 9301, Free State, South Africa; ^6^Department of Veterinary Medicine and Public Health, College of Veterinary Medicine and Biomedical Sciences, Sokoine University of Agriculture, P.O. Box 3021, Morogoro, Tanzania

## Abstract

Foot-and-mouth disease (FMD) is a highly devastating viral disease affecting all cloven-hoofed animals. The disease threatens food security and livelihoods across different parts of the world. FMD is endemic in Africa; where the continuous circulation of the disease impacts the livelihoods of pastoral communities by reducing the quality and quantity of livestock products such as milk and meat, as well as undermining the access of the livestock sector to regional and lucrative global markets. Strategies used to control FMD in Africa, especially tropical Africa, are typically fragmented national-level focused activities with relatively poor outcomes, rather than regionally coordinated initiatives that have been used on other continents (South America, Europe) to successfully reduce and even eliminate virus circulation. Biotechnological advances have improved our ability to detect and characterize FMD virus strains, but more effective approaches to disease control are needed to encourage disease reporting and outbreak investigation. This review of the challenges to FMD control amongst Africa's diverse pastoral communities is intended to provide information and provoke discussion to improve the strategies and approaches for regional FMD control in Africa.

## 1. Introduction

**Figure 1:**
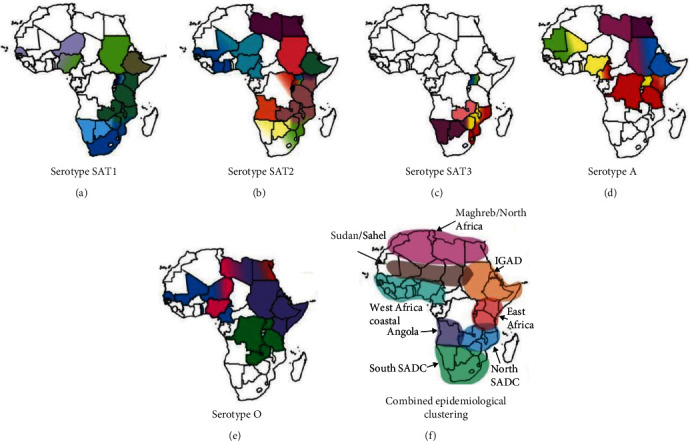
Maps of Africa (a–e) showing the FMDV serotypes and topotypes (color coded) distribution together with the conjectured epidemiological clusters (f). The epidemiological clusters and the color-coded topotypes shown in maps (a–f) do not necessarily show legitimate political borders of the countries. Abbreviations: IGAD, Intergovernmental Authority on Development; SADC, Southern African Development Community; SAT, Southern African territories [6].

Foot-and-mouth disease (FMD) is a highly contagious disease that affects all cloven-hoofed livestock and wildlife animals, with impacts on food security and the socioeconomics of livestock-dependent communities [1]. The disease is caused by foot-and-mouth disease virus (FMDV) that belongs to the genus *Aphthovirus* of the family *Picornaviridae* [2, 3]. There are seven serotypes of FMDV, namely O, A, C, Southern African Territories (SAT)-1, SAT-2, SAT-3, and Asia-1, five of which have been reported circulating on the African continent (Figure 1(a)–1(e)) [4]. In Africa, FMDV circulates in three main virus pools or ecosystems [5], subdivided further into eight epidemiological clusters (Figure 1(f)) that are dynamic rather than fixed, requiring regular review and updating [6]. FMD is a potential transboundary animal disease (TAD) that requires properly coordinated national, regional, and global progressive control strategies [7–9]. The disease is a major obstacle to both commercial and traditional livestock production systems and even has impacts on the continent's wildlife sector [10, 11].

FMD has a long history in Africa, and countries have struggled for many years to prevent, control, and eliminate the disease through various strategies and initiatives [12]. FMD epidemiological knowledge in livestock and wildlife populations provides vital evidence to design control strategies [13, 14]. Zoo-sanitary measures, such as quarantine and movement permits and the use of physical fences to separate wildlife from domesticated species, are critically important to break the cycle of infection for highly contagious diseases like FMD. These may be supplemented with vaccination and culling of affected herds, though culling may be appropriate in the final stages of eradication. Conversely, in parts of Africa where FMD is more common and where pastoralists predominate, vaccination has always been regarded as the main FMD control approach [10], since control of animal movements is difficult and there are usually insufficient resources to compensate owners in support of livestock culling. However, vaccination is a complex task, requiring a sound strategy, proper implementation, and regular review [15], in order to be effective, especially where complementary control measures are weak [16, 17]. Where resources are available, new tools can be used for partial genome and whole-genome sequencing of FMDV [18, 19], as well as for serological surveillance using commercial kits. Although these biotechnological advances help to understand the epidemiology of FMD (Figure 1(a)–1(f)), FMD remains a regional and global problem with significant food security and livelihood impacts that affect vulnerable communities.

Surveillance activities continue to monitor the occurrence, emergence, and spread of different FMDV serotypes and lineages, including antigenic characterization, to inform vaccine selection [5] (Figure 1(f)). This knowledge can help direct control efforts properly [20, 21]. In the last 10 years, studies have started to investigate the African pastoral context in which control measures must operate and to consider how social and disease control measures can be reconciled [22]. This review explores the FMD control challenges related to the highly diversified pastoral communities [23] and their implications for the improvement of FMD control strategies across Africa.

## 2. Africa's Pastoral Systems and Its Impact on FMD

The livestock sector in Africa is predominated by pastoral and agropastoral systems [24]. Pastoralists are found in all regions of Africa with characteristic variable livestock husbandry practices that pertain to their traditional and cultural systems but are influenced by geography, topography, and climatic conditions [25, 26]. According to Robinson [27], there are five main livestock husbandry practices identified in Africa that operate between pastoral and agropastoral systems, namely (i) total nomadism (no permanent place of residence and no regular cultivation), (ii) seminomadism (a permanent place of residence exists and supplementary cultivation is practiced, but for long periods of time animals travel to distant grazing areas), (iii) transhumance (a permanent place of residence exists, and herds are sent to distant grazing areas, usually on seasonal cycles), (iv) partial nomadism (farmers live in permanent settlements and have herds at their disposal that graze in the vicinity), and (v) stationary or sedentary animal husbandry (animals remain on the holding in the village throughout the year). In most cases, more than one livestock husbandry practice operates in a particular geographical area, and each has distinct animal movement characteristics [28, 29]. Pastoralists in Africa are unevenly distributed in the savannah lands and experience diverse climatic conditions across the continent. Some of the areas experience floods of varied duration and rivers that are challenging to cross in some seasons [30, 31]. The pastoralists in Africa are unevenly distributed in the savannah lands and experience different climatic conditions across the continent [28, 29]. Some of the areas experience floods of variable duration and rivers that are difficult to cross in some seasons [30, 31]. Pastoral communities that are diverse and always dynamic [32, 33] make FMD surveillance and monitoring activities challenging due to difficulties to trace/track animals [34–36]. Maintenance of FMD infection in these pastoralist settings provides opportunities for spread between herds and is thought to influence the regional virus circulation patterns [6]. The disparities and complexity amongst pastoralist practices across Africa indicate that tailored approaches will be required to achieve FMD control.

## 3. Tradition Practices of Farming Multiple Species

**Figure 2:**
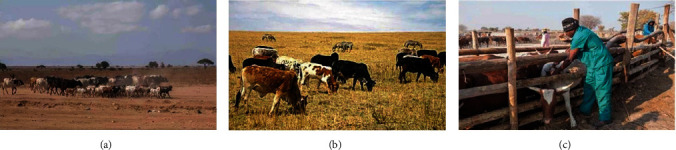
(a) Mixed species of small and large ruminants (goats and cattle) kept together between Dodoma and Morogoro, Tanzania (Source: David Paton, 2019). (b) Livestock and wildlife cograzing during the drought season at the Serengeti ecosystem interface (Source: This study, 2017). (c) Cattle vaccination for FMD in Namibia (Source: David Paton, 2015).

Unlike highly commercialized farms in many other parts of the world, most of pastoral communities across Africa such as the Maasai in Kenya and Tanzania often keep multiple species of FMD-susceptible livestock [23] including cattle, sheep, and goats [37, 38]. There is uncertainty over the role of some species in the epidemiology of FMD, which likely depends on stocking density and contacts with other species (Figures 2(a) and 2(b)). The tendency of pastoralists to value and care for cattle more than other species also has implications for FMD control strategies. Similarly, cattle have been the main focus of scientific research and veterinary service provision, such as vaccination programs [15, 39] with other animal species often neglected. This underlines knowledge gaps on the relative contribution of different species in the epidemiology of FMD [8, 40].

## 4. War and Conflicts in Africa Pastoral Communities

Pastoral communities commonly experience conflicts that endanger human and animal lives [41, 42]. Four key categories cover most reported conflicts. The first category involves struggles within pastoral communities [32, 43]; the second involves struggles between pastoralists and the farming communities [44, 45]; the third involves pastoralists being affected by rebellion groups [46, 47]; and the fourth is between pastoralists and government authorities [45, 48]. Access to grazing lands and pastures, water sources, and animal theft often lead to conflicts [49–51]. These problems are all exacerbated by governance problems [52]. In Africa's FMD-endemic areas, civil wars followed by protracted postwar recovery and stabilization have been witnessed [53], and this can often lead to a lack of permanent settlements for pastoralists. The free-roaming pastoral communities distort social programs like vaccination campaigns [32]. Wars and conflicts foster food insecurity, diseases, and vulnerabilities, particularly poverty [23, 54] making animal disease control initiatives a low priority. The disputes in Kenya (al Shabaab, postelection unrest), Uganda, South Sudan, the Central African Republic, the Democratic Republics of the Congo and Chad (Lord's resistance army), and Nigeria (Boko Haram) [46, 47, 55] highlight the type and extent of unrest in Africa. In incidents like these, animal health service providers hesitate to travel to remote areas for fear of their security, hence paralyzing animal health programs. Rweyemamuet et al. [56] revealed how insecurity prevented surveying the Southern Somali ecosystem and implicating timely release of global rinderpest eradication declaration reports.

## 5. Dissimilar Policies on Pastoral Undertakings across African FMD Endemic Countries

Pastoralists in Africa experience a diverse range of policies from their respective governments [57, 58]. For example, Kenya recognizes pastoralism and provides some safeguards as stipulated in the Community Land Act No.27 [59]. In Tanzania, there is no land legislation specific to pastoralism [54] even the National Land Policy of 1995 [60] does not cover pastoral activities in relation to their land resource use and ownership [61]. In Zambia, most land is held by tribal chiefs and managed in common [62–64]. Land policy in Botswana differs most, with predominantly private ranches for livestock production [65, 66]. Increase in human population with growing demands for cultivation and mining activities are evident in Africa as elsewhere [67–69] and have exerted pressure on many pastoral communities, which often do not have legal papers for land ownership [55, 58, 70, 71]. This pressure has resulted in an increased migration of pastoral communities to areas where they can access land for readjustment of their pastoral activities [72]. These differences in national policies and circumstances call for tailored and strategic approaches to deal with animal health problems. Achievement of TADs control (FMD inclusive), therefore, requires an understanding and adjustment of underlying policies that impact pastoralists and their practices.

## 6. Animal Movements and FMD Epidemiology in African Pastoral Settings

Most pastoralists keep livestock for prestige and livelihood sustenance. The key reasons for movements of animals involve communal grazing land, transhumance, oxen for transport, bulls for mating, dowry transfers, arable land cultivation, and for other social actions like refraining from unsettled disputes. Furthermore, pastoral communities migrate animals due to excessive drought and rains that destroy habitats, pastures, water sources, and crops [73]. The lack of priority given to pasture management necessitates finding new pastures and water sources [74, 75]. Animal movements influence livestock–wildlife interactions, providing opportunities for subsequent interspecies transmission, and spread of infectious disease pathogens [1]. Kangalawe et al. [76] provided background describing pastoral and agropastoral community movements from the early 1950s in Tanzania, mostly involving Maasai, Barbaig, and Sukuma peoples. The authors also cited the rise in north-to-south migration to Mbeya, Iringa, and other regions of Kigoma, Rukwa, Morogoro, Pwani, and Rufiji that occurred in the 1980s. The primary drivers were gradual climatic changes, soil or land degradation from overgrazing and overstocking, poor agricultural practices, and population increase in their areas of origin. In Kenya, predominant pastoral communities are Maasai and Turkana, which have limited mobility due to stringent Kenyan government land policies adopted from the British colonial era [77]. In West Africa, Fulani pastoralist groups in the Savannah and Sahel rangelands operate regulated transhumance movements under the Economic Community of West African States (ECOWAS) 1998 Protocol on Transhumance [78] to monitor of risks for animal diseases epidemics like FMD.

Unmonitored movements have potential to spread the FMDV to susceptible hosts, thereby undermining control efforts [79]. For instance, the 2018 FMD serotype O outbreaks that started in Algeria and spread to Tunisia and lastly Morocco in January 2019 were genetically closely related to the O/EA-3 topotype that originates from West Africa. Furthermore, serotype A outbreaks in Algeria in 2017 FMD were genetically closely related to the Nigerian FMDVs [80]. Similarly, the 2006 FMD serotype A outbreaks in Egypt were genetically closely related to the East Africa topotype (G-VII genotype) that had already been reported in Kenya and Ethiopia [81]. Recently, serotypes O and A have spread southward from East Africa to Zambia [82], Malawi, and Mozambique. Animal movements render vaccination campaigns challenging though missed or incomplete vaccination and reduce the efficiency of disease control programs [15]. African animal health authorities deploy three epidemiological strategies to control animal movements. The first is quarantining, which aims to prevent infected animals from infecting nearby herds. The second is issuing movement permits that are approved after acquiring health and vaccination certificates for regulating long-distance movements of animals. Unfortunately, dishonest officials and pastoralists can misuse these permits, reducing their impact to control animal diseases [35]. The third option is based on controlling local movements, e.g., transhumance through the identification of defined areas within which free movements can occur. Animal health and law enforcement officers have to identify target animal herds under all movement control settings and do monitoring (http://www.fao.org/3/w3737e/w3737e12.htm). Some countries such as South Africa, Botswana, Namibia, and Zimbabwe have also erected physical fences to curb animal movements. Game-proof fences restrict both livestock and wild animal movements and their interactions [83]. However, fencing is expensive and creates socioeconomic and environmental concerns and interrupts wildlife migration, and these may outweigh the FMD control benefits [84, 85]. Although fencing has not been widely adopted by other African countries facing similar FMD problems, animal identification is also poorly practiced in most of Africa's FMD-endemic areas.

## 7. Wildlife Involvement in the Epidemiology of FMD in African Pastoral Settings

Many African countries contain wildlife conservation areas that possess rich pastures and water sources [86]. Most pastoral communities are settled around these conservation areas and strategically graze their animals within the parks during droughts, despite the stringent restrictions imposed by the government authorities [87–89]. Studies have shown that SAT serotypes of FMDV are maintained in African buffalo herds (*Syncerus caffer*) for 24 years or longer [90]. Moreover, transmission of FMDV from infected buffalo in direct contact with cattle can occur under certain conditions of livestock–wildlife interaction, providing a mechanism by which new variants can be introduced into livestock within and around protected areas (Figure 2(b)) [87, 91]. Evidence of long-term FMDV maintenance through virus persistence in infected wildlife of species other than the African buffalo has not been shown, and more efforts are needed to define the FMDV host spectrum in wildlife and their roles in virus spread and maintenance. Such work is hampered by the practical difficulties of conducting surveillance studies in wildlife essentially due to high expenses [92, 93]. The presence of FMDV persistently infected wildlife complicates the epidemiology of the disease and its eventual eradication prospect [88, 94].

## 8. Challenges of FMD Surveillance Systems in African Pastoral Settings

Surveillance is essential to understand the risks posed by animal diseases [35, 95]. These activities employ active or passive surveillance to monitor FMDV circulation and identify new virus in the field [6] including high-risk areas, such as the livestock–wildlife interface [35, 87, 94]. Surveillance is expensive since it requires data collection at regular intervals and proper record keeping. Obtaining relevant data on TADs like FMD is difficult as record keeping is a challenge in most Africa countries, though it requires effective coordination of pastoralists at all levels [34]. However, pastoral communities live in isolated, scattered, remote, infrastructure-poor, and underdeveloped areas [96], and some in cross-border ecosystems to access communal grazing areas [97, 98]. At certain periods of the year, livestock and their owners become unavailable for surveillance and disease control programs. Thus, considerable flexibility, commitment, time, patience, and passion are needed for retrieving information when conventional approaches are deployed. Among the different surveillance studies employed in Africa, participatory surveillance remains a cost-effective method of gathering vital data from pastoral communities, especially in remote, FMD high-risk pastoral areas [99]. However, the lack of proper knowledge and commitment to record keeping (i.e., vaccination, vaccines, outbreaks, etc.) for the pastoralists and other key players in diseases control is a notable drawback. The lack of appreciable immediate benefits on animal health surveillance activities exacerbates these challenges [89] especially since pastoral communities have little interest in FMD control programs as they do not appreciate the benefits that may accrue from its successful control [11]. The FMD risk-based animal movement epidemiological surveillance data generated should help to implement vaccination program tactics to improve FMD control effectiveness [100]. Interestingly, most FMD surveillance data from pastoral communities in Africa is generated by research studies rather than routine operational surveillance systems. Such studies are infrequent and need considerable time, resources and expertise to design, implement, and analyze for a specific intended purpose.

## 9. The Burden of Other Diseases with Human and Animal Health Implications

Apart from FMD, African countries and, in particular, the pastoral communities face significant challenges from other diseases with serious human and animal health consequences [101–103]. Based on the WOAH classification, 15 diseases are considered to be the most contagious and of these, 12 are found in Africa [52]. In the FAO Emergency Centre for Transboundary Animal Diseases reports, it is estimated that about 90% of the WOAH-listed diseases are known to occur in Tanzania [104]. The black-quarter (BQ) [105], contagious bovine pleuropneumonia (CBPP) [106], contagious caprine pleuropneumonia (CCPP) [107], peste des petits ruminants (PPR) [108], and East Coast fever (ECF) [109] are among highly reported animals diseases in different African geographical locations. Considering that over 60% of human diseases are of animal origin, the close association of the pastoral communities with their animals increases their risks of zoonotic infection [110] when compared to other communities, bearing in mind that over 60% of human diseases are of animal origin [111, 112]. Some of the most commonly reported human diseases are brucellosis [110], anthrax [113], tuberculosis (TB) [114], HIV-AIDS [115], rabies [116], malaria [117], cholera [118], and rift valley fever and salmonellosis [119]. New disease(s) epidemics like Ebola [120] further drain resources [121]. Most African countries have weak economies [122] and prioritize the control of human diseases over animal health. The future risks and threats to neglected human and animal diseases are expected to escalate due to changes in trends of disease drivers [52], putting extra strain on budgets for control of TADs. Most African governments have a tendency to express high sensitivity and greatest concern for diseases that cause massive mortalities [123] and condemnation of products. The indiscriminate use of unprescribed drugs in Africa intensifies animal and human health threats from antibiotic-resistant pathogens [52]. These are the reasons why FMD control initiatives in most African countries have hitherto been sluggish and unsuccessful. The budgetary constraints and unsustainable commitment have rendered control efforts on FMD and other TADs as temporary, contrary to the rinderpest eradication program which earned global-level commitment [52].

## 10. Vaccine Performance and Vaccination Challenges in African Pastoral Settings

In FMD endemic regions, vaccines can be used to protect high-value animals, such as dairy cows, a strategy advocated for countries at the early stages of the progressive control pathway for FMD (PCP-FMD) [15]. The FMD vaccines available in Africa and elsewhere consist of whole virus particles, chemically inactivated with binary ethylene-imine (BEI) and combined with oil-based or aqueous adjuvants [124, 125]. These vaccines induce short-lived immunity and require booster vaccinations in order to provide sustained protection [39, 126]. Therefore, maintaining immunity over time needs repeated vaccinations (prophylactic vaccination) or by one-off vaccination to provide temporary protection against a specific threat such as nearby outbreaks (reactive or emergency vaccination). For prophylactic purpose, naïve animals should get two initial vaccinations 3–4 weeks apart, followed by revaccination at every 4–6 months. FMD control requires a regular vaccination of whole populations within a given zone and measures to prevent virus incursions, especially from animals outside the zone. Unfortunately, due to limited resources, most FMD-endemic countries fail to prioritize vaccination and complement it with other control measures. Under African states, some manufacturers recommend five vaccinations per annum, depending on the vaccine potency [124, 125, 127] and FMDV challenge weight [15]. Unfortunately, pastoralists cannot afford multiple revaccinations annually [10, 128]. Vaccines sourced from the Kenya Veterinary Vaccine Production Institute (KEVEVAPI), Kenya, and the Botswana Vaccine Institute (BVI), Botswana, (O, A, and SAT1 and 2) cost about 1.60 USD and 2.24 USD per single dose in Tanzania, excluding the logistical costs [129].

A key point with FMD vaccines is that they are thermally sensitive and require intensive cold chain handling to maintain their good quality from leaving the manufacturer until reaching scattered pastoralists in the field [15]; this also has cost implications [10]. Most herders lack permanent physical addresses or settlements making planned vaccination(s) (Figure 2(c)) and postvaccination evaluation(s) challenging [33, 51]. Pastoral communities are insufficiently educated [130, 131] making it more difficult to raise awareness that complement vaccination with other control measures, like biosecurity precautions. For these reasons, emergency vaccination is the often preferred strategy for government intervention. However, this approach requires good surveillance to define the vaccination zone, a rapid response with potent vaccines, good biosecurity to prevent the vaccination teams from spreading infections, and controls on infected livestock being moved beyond the vaccination zone. Additionally, African vaccines are rarely tested or checked for their quality or strain match, and their field performance is seldom monitored or critically reviewed. The Africa Union-Pan African Veterinary Center of the African Union (AU-PANVAC) has a mandate to check the quality of vaccine batches used in African livestock, but this is not yet done as sourcing, immunization, challenging, and monitoring of naïve cattle with the FMDV has been major drawback. In the absence of government support, pastoralists may be obliged to pay for their own vaccine and plan their own vaccination schedules which is difficult without proof of cost–benefit, guidance, and coordination [6].

## 11. Status of Infrastructures Required for FMD Control in African Pastoral Settings

The infrastructure networks and social services necessary for diseases control (roads, electricity, water, banks, veterinary clinics, veterinary laboratories, hospitals, and schools) in most pastoral communities are underdeveloped [132]. Furthermore, the presence of diverse types of obstructive geographical features (rivers, valleys, hills, and steep mountains) in these highly dispersed communities complicates logistics further and increases transport, materials storage, vaccination, and monitoring expenses for disease control [6, 133]. Due to resource constraints, most of the government authorities in Africa give a low priority to disease control in pastoral areas. This makes effective provision of services in these areas even more challenging [130, 134]. Even competent animal health personnel can become discouraged from working in pastoral areas due to this challenge [135]. Therefore, it is difficult for any animal health program (e.g. vaccination programs) to reach pastoral communities in a timely manner and this impacts negatively on the effectiveness of the FMD control programs [6]. Furthermore, the one health approach needs to be emphasized to enable sharing of some pertinent missing services or facilities in remote areas. And finally, the government(s) need to undertake some land policy reforms to accommodate and transform pastoral activities, and suit infrastructures access that facilitates animal diseases control.

## 12. The Difference in Animal Disease Control Priorities among African Countries and Communities

Despite many similarities in the challenges from diseases of human and animal health [101, 102], there are marked differences in priorities for control of FMD and other endemic livestock diseases between communities and countries [136]. For example, a participatory study in the Maasai Mara ecosystem in Southwestern Kenya revealed that pastoralists had different ideas about which animal diseases were most important and how they affected their lives [137]. According to pastoralists who participated, FMD has the highest impact on milk production and is ranked second to CBPP based on ascribed losses instead of ECF. In Tanzania, CBPP is regarded as the highest priority disease among all frequently reported livestock diseases (ECF, trypanosomosis, CBPP, BQ, TB, and anthrax) [138, 139]. In Uganda, FMD stands first in their priority lists for disease control. The economic disparities between countries affect the disease control priorities; for instance, low-income countries experience high challenge of infectious zoonotic disease burden of about 13%, as compared to 1% in high-income countries [136]. The countries with many cattle are likely to be more enthusiastic and ambitious to export animal products to lucrative markets after controlling FMD, but this may be offset by the lack of resources, expertise, and priorities, contrary to the outlook in Southern Africa countries like South Africa and Botswana. Currently, there is no single disease that African countries have decided to control strategically by joint efforts /initiatives under regional agreements as stipulated in the OIE: FAO's [36] document. This lack of harmonization in rankings creates difficulties in agreeing which diseases should be prioritized for control, particularly where joint initiatives among African countries are needed. Perhaps there are lesson to be learnt from the 2011 global Rinderpest eradication success, where all countries were fully engaged in the campaign process [140].

## 13. Possible Options for Improving FMD Control under the Current Pastoral Settings in Africa

**Figure 3:**
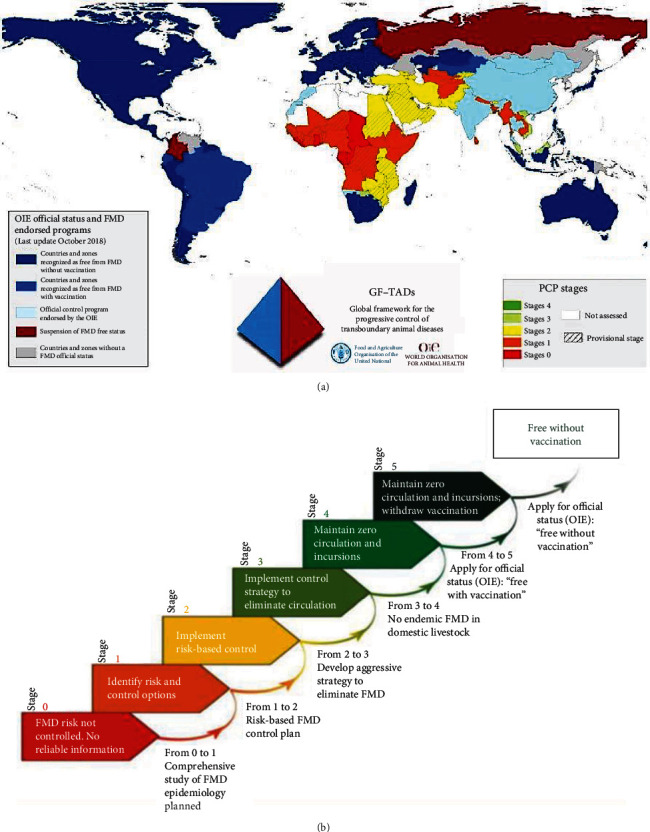
(a) Global map showing countries PCP-FMD stages, their respective OIE official statuses and corresponding endorsed programs on FMD control (OIE and GF-TADs, 2018). (b) The summarized stages for the progressive control pathway for FMD control (PCP-FMD) are applicable to all countries endemic to FMD (OIE and GF-TADs, 2018).

FMD control in the African endemic context faces multiple challenges that need to be addressed systematically to convey tailored solutions concepts. FMD control priorities vary and most countries are in PCP-FMD stages 1 and 2 with only a few in stages 3 and 4 (Figures 3(a) and 3(b)). The FMD free countries avoid new FMDV incursions that can lead to suspension of their FMD freedom status and beef exports. These countries are challenged by new FMDV variants, maintaining adequate vaccination immunity, and managing biosecurity measures at FMD high-risk areas like livestock–wildlife interfaces and pastoralists' animal movements. They address animal introduction prevention, prophylactic vaccination, and active monitoring for early detection and rapid response to FMD incursions [9, 141]. The countries at early stages are mostly challenged by the availability of resources, often because of the absence of export markets. PCP-FMD can be executed on a single-country basis but due to porous borders, joint actions are emphasized to enhance outcomes (Figures 3(a) and 3(b)). The countries can progress together by (1) ecosystem-based approaches as described by Maree et al. [6], (2) geographical-based approaches via defined regions in the continent (East, Central, West, South, and North) or as a whole continent, as in Europe and South America under EuFMD and COLSAFA, respectively, and (3) political-based approaches via multiple countries establishing political communities/forums like The Southern African Development Community (SADC), ECOWAS, and East Africa Federation (EAF).

Sustainable regional and inter-regional trade in Africa needs commodity-based trade (CBT) and other nongeographic FMD control approaches [142]. The latter strategy emphasizes removing the risk of infection from final products or commodities, despite whether the infection has been eradicated from a region's entire livestock population or not. For example, a hazard analysis critical control point may be used to destroy FMDV during beef processing (beef maturation after deboning and lymph nodes removal). Biosecurity measures and vaccinations can be enhanced to protect livestock against FMDV infection in established compartments [142]. Thus, CBT reinforces the 2012 Phakalane Declaration on trade of beef from places where FMD cannot be easily eliminated by the available geographical-based control measures. The integration of movement controls, vaccination strategies, and biosecurity measures in an African pastoral context could minimize infections, and strengthen animal products trade [142]. The adoption of value chain-based approach to CBT when merged with participatory-based studies [143–145] could promote pastoral communities participation to minimizing FMD contamination risk.

During rinderpest surveillance programs in Pakistan and Kenya, pastoralists helped identifying priority disease in various geographical locations to promote pastoralists' prompt response and execution of mitigation measures [146]. Pastoralists can readily adopt disease controls when they experience practical benefits in the livestock production value chain. Adoption of control measures may also aid in managing other diseases that pose a threat to their animals as was the case with HPAI control in Africa [99, 144].

FMDV surveillance programs need to be enhanced to determine spatiotemporal distribution of FMDV strains and to identify FMD risk hotspots based on transmission. The rapid and accurate field deployable diagnostic tools like portable qRT-PCR [147] need to be emphasized to support timely diagnosis during outbreaks. Participatory epidemiology and surveillance programs need to be improved by using digital mobile technologies [148], such as the SACIDS-AfyaData app in Tanzania [149]. This will promote quick access and hasten sharing of data at local, national, and international levels [99]. The mapping of the dominant animal movements needs to be done concurrently to understand animal risk pathways in pastoral regions [142]. With suitable vaccination programs, this will foster opportunities for commercial activities and investment via identified and established FMD-free zones and compartments. Strengthening of public–private partnership approaches to improve FMD vaccine value chain awareness from production, purchasing, distribution, delivery, vaccination, and postvaccination monitoring to FMD endemic and pastoralists predominated countries will enable effective vaccine usage and performance [150].

The countries need to enhance laboratory capacity and networks to diagnose and characterize FMDV in a timely manner. A primary driver should be to encourage countries with unknown FMD status to submit outbreak samples and reports. Laboratories need to take part in FMD mitigation quality control activities like vaccine selection and vaccination monitoring, use of high potency vaccines matched with field circulating strains, recommended vaccination schedule, and coverage are required. Finally, regional strategies and initiatives to control FMD in Africa should be coordinated in multicountry state rather than being centered on individual country efforts, with animal health policies adjusted to accommodate existing disparities among FMD endemic countries [99].

## 14. Conclusion

This review highlights FMD control challenges across Africa in the context of pastoral communities. African pastoral practices need to be perceived as historical adaptations to survive difficulties along with changing climatic conditions rather than a nuisance. This review reveals how pastoral communities operate in particular ecological contexts that render FMD maintenance and control across Africa more complicated than in any other regions in the world. This calls for tailored mitigation approaches that address:

- Reform of land policies in FMD endemic countries to suit sustainable environmental management practices and enhance pasture and water availability. This will also support the establishment of FMD control infrastructures and promote opportunities for PCP-FMD progress.
- The lessons of cooperation via FMD control strategies in Europe and South America. Initiate an AU-FMD platform to foster regional FMD scientific studies, present evidence needed to accelerate CBT, and catalyze PCP-FMD participation.
- Emphasize ecosystem-based disease control approach to account for unique pastoral animal movements, the multiplicity of circulating FMDV field variants, the limited availability of vaccines, vaccination, and monitoring.
- Enhance participatory surveillance systems for gathering pastoral knowledge with quick access and multisectoral sharing of information to improve preparedness and rapid response to epidemic diseases like FMD.
- Countries need to consider FMD vaccines as a public good or subsidize, demonstrate their cost-benefits to promote appropriate uptake by pastoralists, and ensure availability of suitable quality vaccines and indicate where they can be effective. In cases where vaccination campaigns experience adequate financing, then the private sector may have to take responsibility for vaccination, as for other endemic diseases.
- The WOAH guidelines (TAHC Chapter 4.4 and Chapter 4.5) for FMD free zones and compartments (OIE, 2014) facilitate exports to FMD free countries, but there is a gap in guidance on appropriate risk mitigation for trade between infected countries and zones, and there are no WOAH guideline for CBT that could accelerate trade amongst FMD challenged countries with roaming pastoral herds and carrier wildlife [87, 88, 142].

The future studies of animal movements across African need to be combined with molecular epidemiological data generated from circulating FMDV strains to improve outbreak predictions and proper vaccine usage. Also, the future vaccines for Africa need to consider thermostability, protection duration, and cross-protection challenges for improving logistical and vaccination expenses.
